# Comparative proteome analysis revealed the differences in response to both *Mycobacterium tuberculosis* and *Mycobacterium bovis* infection of bovine alveolar macrophages

**DOI:** 10.3389/fcimb.2023.1266884

**Published:** 2023-11-01

**Authors:** Yurong Cai, Weifeng Gao, Pu Wang, Gang Zhang, Xiaoping Wang, Lingling Jiang, Jin Zeng, Yujiong Wang, Zhiwei Wu, Yong Li

**Affiliations:** ^1^ Key Laboratory of Ministry of Education for Conservation and Utilization of Special Biological Resources in the Western China, Ningxia University, Yinchuan, China; ^2^ School of Life Science, Ningxia University, Yinchuan, China; ^3^ The Fourth People’s Hospital of Ningxia Hui Autonomous Region, Reference Lab, Yinchuan, China; ^4^ Center for Public Health Research, Medical School, Nanjing University, Nanjing, China

**Keywords:** autophagy, bovine tuberculosis, *Mycobacterium tuberculosis*, inflammatory response, resistance mechanism

## Abstract

Tuberculosis (TB), attributed to the *Mycobacterium tuberculosis* complex, is one of the most serious zoonotic diseases worldwide. Nevertheless, the host mechanisms preferentially leveraged by Mycobacterium remain unclear. After infection, both *Mycobacterium tuberculosis* (MTB) and *Mycobacterium bovis* (MB) bacteria exhibit intimate interactions with host alveolar macrophages; however, the specific mechanisms underlying these macrophage responses remain ambiguous. In our study, we performed a comparative proteomic analysis of bovine alveolar macrophages (BAMs) infected with MTB or MB to elucidate the differential responses of BAMs to each pathogen at the protein level. Our findings revealed heightened TB infection susceptibility of BAMs that had been previously infected with MTB or MB. Moreover, we observed that both types of mycobacteria triggered significant changes in BAM energy metabolism. A variety of proteins and signalling pathways associated with autophagy and inflammation-related progression were highly activated in BAMs following MB infection. Additionally, proteins linked to energy metabolism were highly expressed in BAMs following MTB infection. In summary, we propose that BAMs may resist MTB and MB infections via different mechanisms. Our findings provide critical insights into TB pathogenesis, unveiling potential biomarkers to facilitate more effective TB treatment strategies. Additionally, our data lend support to the hypothesis that MTB may be transmitted via cross-species infection.

## Introduction

Tuberculosis (TB) is an enduring infectious disease that has significantly impacted human and animal populations for centuries, at one time leading to a high number of fatalities and earning the moniker, the "white plague." In the contemporary era, TB remains one of the most serious infectious diseases worldwide, and its impact has increased because of increased population mobility (Seung et al., 2015). This grave disease is caused by chronic infection with the *Mycobacterium tuberculosis* complex (MTBC), which includes *Mycobacterium tuberculosis* (MTB) and *Mycobacterium bovis* (MB). These pathogens can infect both cattle and humans (Daley, 2010). MTB, which primarily infects humans, is highly infectious but does not pose an immediate existential threat to its hosts. In contrast, MB possesses the capability to infect various host species and maintain an infectious cycle (Whelan et al., 2010; Corner et al., 2012), demonstrating higher virulence (Palmer et al., 2012). Hence, tuberculosis not only impedes the advancement of the cattle breeding industry but also poses serious threats to human health and life. A global survey disclosed that in 2016 there were 10.4 million new tuberculosis cases, of which approximately 10% were attributed to MB infection in humans (Waters and Palmer, 2015; An et al., 2017). Therefore, the effective containment of bovine tuberculosis may be an effective strategy to mitigate the risk of human TB infection. However, the mechanisms underlying the preferential hosts of MTBCs remain unclear.

During an MTB infection, a host exhibits a distinct immune response to the pathogen (Ouyang et al., 2016). Given that cattle can be hosts of MTB infection, they are ideal large animal models for TB research. As the capacity to infect macrophages is vital for the transmission and propagation of pathogenic bacteria within a host, the interaction between the MTBC and macrophages has become a focal point in studying anti-TB immune mechanisms. Macrophages, functioning as immune regulatory and effector cells, orchestrate the body's inflammatory and immune responses via phagocytosis, antigen presentation, and secretion of various cytokines during the infection process. These cells are important immune cells in the body and play important roles in host anti-infection responses (Hmama et al., 2015; Ouyang et al., 2016). MTB and MB as typical intracellular parasites, macrophages can provide them with nutrients and places to survive and multiply, and they can eliminate them through apoptosis or autophagy (Boddu-Jasmine et al., 2008; Moraco and Kornfeld, 2014; Pawar et al., 2016). It is well established that MTB can prevent phagosome acidification and fusion with lysosomes, thereby evading proteolytic enzyme hydrolysis and subsequent immune responses, which is a principal strategy for MTB to circumvent host cell clearance (Wynn et al., 2013; Ke et al., 2020). In recent years, studies of MB have shown that it can also escape immunity by this strategy (Canaday et al., 2001). Several studies have indicated that MB infection can trigger abnormal expression of long noncoding RNA (lncRNAs) and mRNAs within the body (Boddu-Jasmine et al., 2008; Pawar et al., 2016; Ke et al., 2020). These differentially expressed lncRNAs participate in the regulation of cell signalling pathways, including the Toll-like receptor signalling pathway (TLR), transforming growth factor-beta signalling pathway (TGF-beta), and Hippo signalling pathway (HPO). Therefore, by studying and observing the interaction between bovine macrophages and MTB, we may elucidated differences in host susceptibility to various MTB strains.

In the immune response process, macrophages play vital roles in combating pathogenic bacterial infections and controlling tissue inflammation progression (Wynn et al., 2013). Through various mechanisms, alveolar macrophages are important defence barriers against infection. It has been clearly proven that macrophages defend against MTB infection via phagosome fusion with lysosomes, antigen presentation to the initiate the immune response, activate Toll-like receptors and macrophage apoptosis, and abrogate the secretion of cytokines. Macrophages degrade phagocytic MTB through acidic hydrolases in lysosomes, thereby killing or inhibiting MTB endogenous growth (Hmama et al., 2015). Simultaneously, as crucial antigen-presenting cells, macrophages can breakdown antigens into immunogenic peptides via endocytosis, leading to the production and release of IFN-γ, which inhibits intracellular MTB propagation (Canaday et al., 2001). In macrophages, the toxicity of reactive oxygen and nitrogen products can be increased to stimulate the formation of phagolysosomes and further inhibit MTB dispersion (Sureka et al., 2009). Furthermore, activated cytokines and factors, including TNF-α, IL-1, IL-6, IL-15, IL-10, IL-12, and NF-κB, play significant roles in the macrophage defence response against MTB infection (Sánchez et al., 2012). Therefore, we propose that macrophages may have a pivotal function in determining bacterial preference of a host.

In this study, our objective was to pinpoint the key differences in macrophage responses to MTB and MB infections by performing a comparative proteomic analysis and to elucidate the mechanisms governing hosts preferred by different mycobacteria. We discovered that both MB and MTB infections significantly impact autophagy- and inflammation-related processes in macrophages. Simultaneously, more signalling pathways associated with autophagy and inflammation were altered by MB infection than by MBT infection. In contrast, MTB did not activate these responses to the level that MB activated them. The findings from our research suggest new potential biomarkers to enhance the effectiveness of TB treatment and provide data that support treatments to interrupt cross-species transmission of MTB.

## Materials and methods

### Bacterial strains and culture conditions

The MTB clinical strains M.tb1, M.tb2, and M.tb3 and *M. bovis* strains Mb1, Mb2, and Mb3 were kindly provided by Dr. Xiaopin Wang, The Fourth People’s Hospital of Ningxia Hui Autonomous Region (Yinchuan, China). All strains were cultured to mid-log phase in Middlebrook 7H9 medium (Becton Dickinson and Company) supplemented with 10% catalase medium (oleic albumin dextrose catalase (OADC); Becton Dickinson and Company) and 0.2% Tween 80 (Bio Top Life Sciences). Cultures were maintained in a biosafety level 3 facility and stored at −80°C. Bacteria were harvested from the culture medium by centrifugation at 3000 rpm for 10 min, washed once in phosphate-buffered saline (PBS; Biological Industries, BI), and resuspended in PBS until the optical density at 600 nm (OD600) was 1, which was equivalent to 3 × 10^8^ bacteria/ml.

For the colony counting assay, bacteria were serially diluted 10-fold with Middlebrook 7H9 medium, and 100-µL aliquots of bacteria at each dilution level was transferred to Middlebrook 7H10 (Becton Dickinson and Company) plates supplemented with 10% OADC (Becton Dickinson and Company), 0.5% oleic acid (Solarbio Life Science), and 0.05% Tween 80 (Bio Top Life Sciences). Bacteria were grown for 8-10 weeks until lawn colonies appeared. Bacteria were harvested from the bacterial lawns and resuspended in PBS at an OD600 of 1 to guarantee that the same initial infection dose was applied.

### Cell collection, culture and infection

Primary bovine alveolar macrophages (BAMs) were collected from 3 healthy bovine lungs from cattle 1 to 2 years of age in a beef cattle slaughterhouse in Ningxia (Yinchuan, China). This study was approved by the Ethics Committee for the Use and Care of Animals at Ningxia University (Yinchuan, China). Aseptic sampling of bovinealveolar macrophages was performed immediately after slaughter, and 1 L of saline containing a 4% antibiotic–antimycotic solution (100×) (Solarbio Life Science) was infused into the lungs via the bronchial tubes. The alveolar lavage fluid containing BAM was collected, filtered through a nylon mesh (75 µm), and centrifuged at 1000 rpm for 5 min. The precipitate was resuspended in red blood cell lysis buffer (Solarbio Life Sciences), lysed at room temperature for 10 min and centrifuged at 1000 rpm for 5 min. The precipitate was washed three times with sterile PBS and finally resuspended in RPMI-1640 medium with 10% foetal bovine serum (FBS, Gibco, Carlsbad, USA) and a 4% antibiotic-antifungal agent, and the primary cells were inoculated at a density of 5x10^7^ into a 15-cm-diameter culture dish and cultured at 37°C with 5% CO_2_. To reduce the toxic effects of the antibiotic-antimycotic on the BAMs, we gradually reduced the amount of antibiotic-antimycotic at 6 h, 12 h, and 24 h of the apposed culture. At 24 h, the medium contained 0.5% antibiotic-antimycotic. After incubation, the medium with nonadherent cells was removed, and adherent cells were washed with 15 ml of PBS prewarmed to 37 °C and dissociated by adding 10 ml of prewarmed Tryple™ Express (Thermo Fisher Scientific, Shanghai, China) to each culture flask. The cells were then spun into sediment (1000 rpm, 5 min) and resuspended in 5 ml of prewarmed RPMI-1640 medium in preparation for cell counting and cell activity assays. The average live-cell recovery per animal was estimated to be ~70%. A total of 3x10^7^ cells were inoculated into a 15-cm diameter cell culture dish and incubated at 37°C and 5% CO_2_ for another 36 hours until mycobacterial infection was induced. The unused cells were frozen and stored (freezing solution: 10 % DMSO (Sigma–Aldrich) and 90 % FBS).

Throughout the duration of cell infection, the medium was removed from the macrophages and replaced with RPMI-1640 medium containing MB 1054 (Mb1), 1060 (Mb2), or 1087 (Mb3) or MTB Gong (M.tb1), Wu (M.tb2), or Zhang (M.tb3) at a multiplicity of infection (MOI) of 10 (10 bacteria per cell). After 6 h, the cells were collected, washed one time with ice-cold PBS and processed for whole-protein extraction. Three independent experiments were performed.

### Sample preparation for label-free proteomic quantification

The sample was sonicated in lysis buffer containing 8 M urea and 1% protease inhibitor cocktail three times on ice using an ultrasonic processor. The debris was removed by centrifugation at 12,000 × g at 4°C for 10 min. Finally, the supernatant was collected, and the protein concentration was measured with a BCA kit according to the manufacturer’s instructions. For digestion, the protein solution was reduced with 5 mM dithiothreitol for 30 min at 56°C and alkylated with 11 mM iodoacetamide for 15 min at room temperature in the dark. The protein sample was then diluted by adding 100 mM triethylamine borane (TEAB) to a urea concentration of less than 2 M. Finally, trypsin was added at a 1:50 trypsin:protein mass ratio for digestion overnight and at a 1:100 trypsin:protein mass ratio for another 4 h of digestion. Finally, peptides were dried via vacuum centrifugation and resolubilized in 20 µl of buffer containing 3% acetonitrile and 0.1% formic acid for subsequent liquid chromatography–mass spectrometry analysis.

### Protein identifcation by LC–MS/MS

The tryptic peptides were dissolved in 0.1% formic acid and directly loaded onto a in-house reversed-phase analytical column. The gradient increase from 6% to 23% solvent in 0.1% formic acid and 98% acetonitrile for 26 min, from 23% to 35% solvent for 8 min and then to 80% for 3 min and holding at 80% for the last 3 min all at a constant flow rate of 400 nL/min on an EASY-nLC 1000 Ultra Performance Liquid Chromatography system. The peptides were subjected to a nanospray ionization (NSI) source followed by tandem mass spectrometry (MS/MS) on a Q Exactive^TM^ Plus (Thermo) mass spectrometer coupled online to the UPLC system. The electrospray voltage applied was 2.0 kV. The m/z scan ranged from 350 to 1800 for a full scan, and intact peptides were detected in the Orbitrap at a resolution of 70,000. Peptides were selected using an normalised collision energy (NCE) of 28, and the fragments were detected in the Orbitrap at a resolution of 17,500. A data-dependent procedure alternated between one MS scan followed by 20 MS/MS scans with 15.0 s dynamic exclusion.

### Data analysis

The MS/MS data were processed using the MaxQuant search engine (vs. 1.6.3.3, Cox and Mann, 2008). MS/MS spectra were searched against the UniProt database concatenated with the reverse decoy database. Trypsin/P was specified as the cleavage enzyme, and up to 4 missed cleavages was allowed. The mass tolerance for precursor ions was set as 20 ppm in the first search and 5 ppm in the main search, and the mass tolerance for fragment ions was set as 0.02 Da. Carbamidomethyl on Cys was specified as the fixed modification, and acetylation and oxidation on Met were specified as the variable modifications. The P value was adjusted to < 5%, and the minimum score for the modified peptides was set to be > 40.

### Subcellular localization prediction

We used woLF PSORT, subcellular localization predication software, to predict subcellular localization. For prokaryote species, the subcellular localization prediction software CELLO was used.

### Gene ontology enrichment analysis

Proteins were classified by Gene Ontology (GO) annotation into three categories: biological process, cellular compartment and molecular function. For each category, a two-tailed Fisher’s exact test was performed to evaluate the enrichment of the differentially expressed proteins compared to that of all the identified proteins. GO terms with a corrected p value (FDR/Q value) < 0.05 were considered to be significantly enriched.

### Pathway enrichment analysis

The Kyoto Encyclopedia of Genes and Genomes (KEGG) database was used to identify enriched pathways. Specifically, two-tailed Fisher’s exact test was performed to determine the enrichment of pathways with differentially expressed protein compared to all the identified proteins. Pathways with a corrected p value < 0.05 were considered to be significantly enriched. These pathways were classified into hierarchical categories according to the KEGG website.

### Protein–protein interaction network

All differentially expressed protein database accessions or sequences were searched against STRING database version 10.1 to identify protein–protein interactions. Only interactions between the proteins in the same searched dataset were selected, thereby excluding external candidates. Interaction confidence in STRING is defined by a “confidence score”; we retained all interactions that had a confidence score ≥ 0.7 (high confidence). The interaction network obtained with STRING was visualised in Cytoscape 3.0.

### qRT–PCR verification of expression of genes encoding autophagy-related proteins

The expression of important defence- and autophagy-related proteins induced by MB infection (Q0VCQ6, Q05204 and Q8HXK9) was investigated through real-time quantitative polymerase chain reactions (RT–qPCR). The specific primers for RT–qPCR were designed using Primer 6 (v6.24) Designer.

Total RNA extraction and quantification were performed as described in Vieira et al. (2016) using an RNeasy Mini Kit (Qiagen, Germany) and a NanoDrop 2000 Spectrophotometer (Thermo Scientific, Waltham, MA, United States), respectively. cDNA was synthesised with a RevertAid^TM^ First Strand cDNA Synthesis Kit following the manufacturer’s protocol (Qiagen, Germany). All reactions were carried out three times as three independent biological replicates. The relative transcript levels of the target genes are presented as the fold change (FC) of the value determined via the 2^-△△Ct^ method. Primer sequences for qRT–PCR are shown in **Table 1**.

**Table 1:** Primer sequences for real-time PCR.

Gene	Primer (Forwards)	Primer (Reverse)
Q0VCQ6	CCGGTCAGTATATGTTTTGTGCT	CATGACTGCATAGAGGGGCA
Q05204	CAACCCCAACAAGACCACCT	GGTCTCGAGAAGCCAAACCA
Q8HXK9	TTTCCAAAGGGCAGACACCC	CACCGTACGCCTCCAGATAG
GAPDH	AACGGATTTGGTCGTATTGG	TTGATTTTGGAGGGATCTCG

## Results

### Quantitative proteomic analysis by label free

To explore the proteomic profile alterations in BAMs following infection with MTB or MB, total protein from three biological replicates of normal BAMs (control), MB- and MTB-infected BAMs were extracted, analysed by LC/ESI-MS/MS and quantified by Label Free. In total, 46018 spectra were generated, and 5467 proteins were identified against the bovine reference.

**Figure 1:**
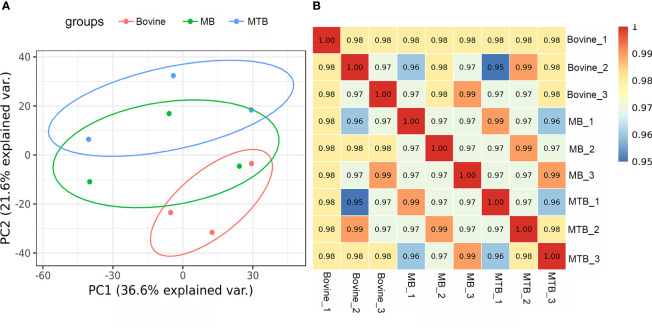
Evaluation of integral proteomic profiles from three treatments. **(A)** Principal component (PC) analysis of protein profiling data of MB-infected, MTB-infected and control BAM groups. Each replicate that received the same treatment is shown in the same plots (red: bovine; green: MB; and blue: MTB). PC1 of the integral proteomic data explained 36.6% of the variance, and PC2 explained 21.6% of the variance. **(B)** Correlation analysis of integral protein data from three treatment groups. The Pearson correlation value is shown for the cells.

The two score plots of the principal component analysis (PCA) models showed a clear separation of samples from different experimental group (MTB and MB groups) and control group BAMs, accounting for 36.6% of the observed variance, indicating that pathogenic bacterial infection was a pivotal factor influencing protein expression (**Figure 1A**). A host cell genotype effect was also discernible, explaining approximately 21.6% of the variance (**Figure 1A**). Notably, the MB-infected samples showed a slightly similar response to that observed in the other two sample groups (the MTB and BAM groups). This suggests that after MB infection, BAMs may exhibit some responses similar to those elicited by MTB challenge (**Figure 1A**). Furthermore, correlation analysis results mirrored the PCA results. The variations in the three biological replicates were calculated based on quantitative data, and minimal variation was found among replicates. This consistency indicates the high quality and reproducibility of the data (**Figure 1B**). Therefore, we propose that both MB and MTB infections led to significant alterations in the gene expression patterns of the BAMs, and similar responses were invoked by infection with either bacterium. The shared and unique responses induced by each pathogenic bacterium type will be the focus of our subsequent investigations.

### Differentially expressed proteins in bovine alveolar macrophages following MTB and MB infection

**Figure 2:**
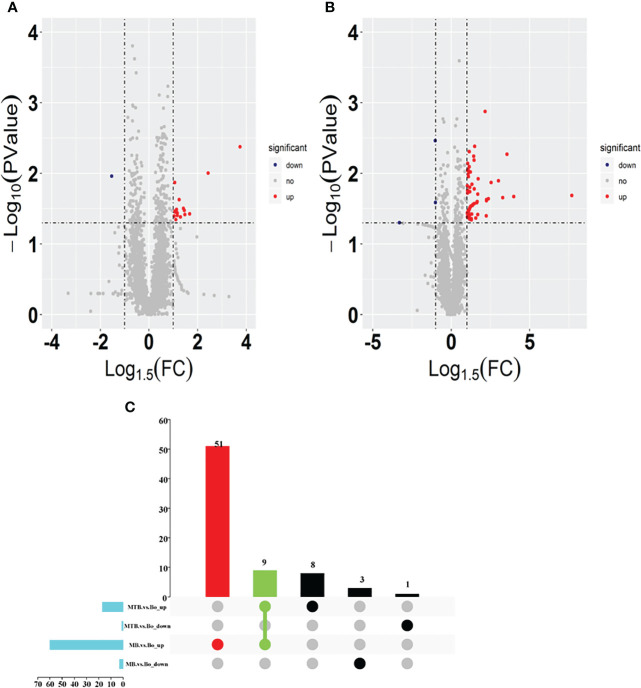
Identification of differentially expressed proteins in both MB vs. BAM and MTB vs. BAM pairwise comparison groups. **(A, B)** Volcano plots showing the potential MTB- **(A)** and MB-induced **(B)** metabolomic features in BAMs. Red points indicate significantly upregulated proteins between the two groups (Log1.5 FC < -1.0 or > 1.0; q-value < 0.05). The blue points show the significantly downregulated proteins between the two groups. The grey points show tentatively matched features with no significant differences. The horizontal and vertical dashed lines represent a P value = 0.05 and FC = 1.5, respectively. **(C)** Venn diagrams representing the overlapping identified differentially expressed proteins in both the MB vs. BAM and MTB vs. BAM pairwise comparison groups; the overlapping proteins were upregulated in any two groups and specifically differentially expressed in MB- and MTB-infected BAMs.

To discern the variations between MTB and MB infection groups, we designed two pairwise comparisons: MTB vs. BAM groups and MB vs. BAM groups. We found that 18 proteins were significantly upregulated or downregulated after MTB infection (**Figures 2A, B**; Log1.5 FC < -1 or >1, P < 0.05). Specifically, 17 proteins were upregulated and 1 protein was downregulated after MTB infection of BAMs (**Figure 2A**). The sole host protein that was downregulated after MTB infection was identified as prefoldin subunit 5 (Q8HYI9). In addition, during MB infection, 60 proteins were upregulated, and 3 proteins were downregulated (**Figure 2B**). The three proteins that showed decreased expression during MB infection were TBC1 domain family member 2A, polysaccharide biosynthesis domain-containing 1, and beta-defensin 10 (A6QP29, F1MV85 and P46168, respectively; **Table 2**).

**Table 2:** Key proteins activated by MB infection.

Protein accession	Protein description	MB/bovine ratio	MB/bovine *P* value	Subcellular localization
**A0A3S5ZPN6**	Scavenger receptor class B member 2	1.688	0.043886	endoplasmic reticulum
**A5PJH7**	LOC788112 protein	1.581	0.03298	extracellular space
**A6H6Y1**	BOLA-DQA1 protein	1.519	0.036096	peroxisome
**E1B726**	Plasminogen	22.512	0.020516	extracellular space
**F1MGW6**	Uncharacterised protein	2.404	0.00133542	cytoplasm
**F1MNI5**	Prostaglandin G/H synthase 2	1.612	0.039212	extracellular space
**F1MX83**	Protein S100	2.48	0.039991	cytoplasm
**F1MYR5**	Nitric oxide synthase	1.615	0.015842	cytoplasm, nucleus
**F1MZL6**	V-type proton ATPase subunit H	1.705	0.037654	cytoplasm
**F1N610**	Ig-like domain-containing protein	1.607	0.031422	extracellular space
**G5E5L8**	Uncharacterised protein	1.665	0.029085	mitochondria
**P09428**	Interleukin-1 beta	4.234	0.005355	cytoplasm
**P26779**	Prosaposin	2.484	0.023632	extracellular space
**P35720**	Succinate dehydrogenase cytochrome b560 subunit, mitochondrial	1.89	0.043107	plasma membrane
**P79345**	NPC intracellular cholesterol transporter 2	1.527	0.018958	extracellular space
**P81287**	Annexin A5	5.042	0.021295	cytoplasm
**Q05204**	Lysosome-associated membrane glycoprotein 1	3.394	0.012726	plasma membrane
**Q0VCQ6**	Programmed cell death 10	1.544	0.008831	cytoplasm
**Q29423**	CD44 antigen	1.62	0.018179	plasma membrane
**Q3SZM3**	Cytochrome b-245 chaperone 1	1.552	0.034538	mitochondria
**Q3T100**	Microsomal glutathione S-transferase 3	1.838	0.004157	plasma membrane
**Q3ZBK5**	Tumour necrosis factor alpha-induced protein 8-like protein 2	2.805	0.013505	cytoplasm
**Q6L708**	Claudin-1	1.564	0.011168	plasma membrane
**Q8HXK9**	Apoptosis-associated speck-like protein containing a CARD	1.531	0.007273	cytoplasm
**Q95123**	Succinate dehydrogenase [ubiquinone] cytochrome b small subunit, mitochondrial	1.736	0.028306	mitochondria
**Q9BGI2**	Peroxiredoxin-4	1.619	0.044665	extracellular space
**F1MV85**	Polysaccharide biosynthesis domain containing 1	0.664	0.025944	cytoplasm
**P46168**	Beta-defensin 10	0.262	0.05	extracellular space
**A6QP29**	TBC1 domain family member 2A	0.662	0.0034376	cytoplasm

“P < 0.05”.

Furthermore, to visualise the proteins that were upregulated or downregulated during MTB and MB infections in BAMs, we created UpSet diagrams, which are depicted in **Figure 2C**. Among the upregulated proteins, nine were upregulated during both MTB and MB infections (**Figure 2C**). Concurrently, 51 proteins were uniquely upregulated in cells with MB infection, and eight proteins were exclusively expressed by MTB infection (**Figure 2C**). These findings suggest that MB infection may stimulate a broader response than MTB infection in BAMs. However, no downregulated proteins were shared between the MTB vs. BAM and MB vs. BAM groups (**Figure 2C**). Therefore, based on these findings, we focused our analysis on three types of upregulated proteins. This helped elucidate the similarities and differences in the responses of bovine cells to MTB and MB infections.

### Functional annotation based on GO and KEGG analyses of nine MTB- and MB-induced upregulated proteins

**Figure 3:**
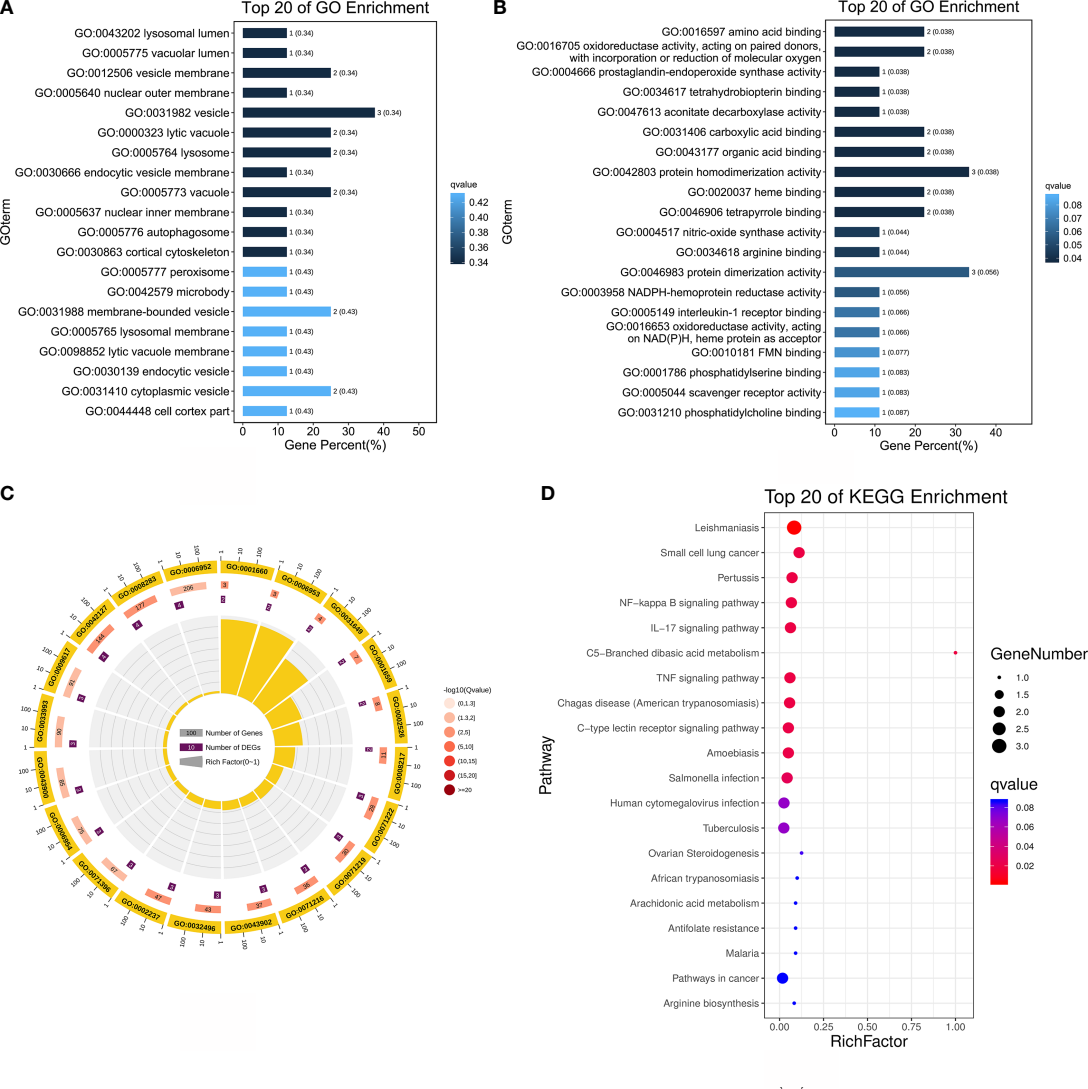
Functional annotation analysis of 9 upregulated proteins after MTB or MB infection. **(A–C)** GO categories enriched with bovine proteins. The proteins were categorised according to GO annotation terms, and the number of proteins enriched in the biological process **(C)**, cellular component **(A)**, and molecular function **(B)** categories is displayed. The number of proteins enriched in each GO category is displayed for each term. The colour of each column represents the significance of each enriched category. **(D)** The pathway enrichment analysis for these 9 significantly upregulated proteins in MB- and MTB-infected BAMs. The scatter plot shows the pathway impact and enrichment results for all 9 significantly upregulated proteins. Each point represents a different metabolic pathway. The various colour intensities indicates different levels of significance of the metabolic pathways from low (blue) to high (red). The different sizes of each point represent the number of proteins involved in the corresponding metabolic pathway. Moreover, the corresponding pathway name of each point is labelled.

To determine whether the nine identified upregulated proteins were significantly enriched in certain functional categories, we conducted Gene Ontology (GO) and Kyoto Encyclopedia of Genes and Genomes (KEGG) enrichment analyses on these proteins (**Figure 3**; **Table 3**). To effectively explore the similarities between the MTB vs. BAM and MB vs. BAM pairwise comparison groups, we initially performed a GO enrichment analysis of the nine aforementioned upregulated proteins (**Figures 3A–C**; **Table 3**). We found that terms in the biological process category were the most significantly enriched, while terms associated with cellular component and molecular function categories were relatively less enriched (**Figures 3A–C**; **Table 3**). Only terms related to the cellular component category, such as lysosomal lumen, lytic vacuole, lysosome, and autophagosome, were enriched in BAMs challenged with MTB or MB (**Figure 3A**; **Table 3**). Previous reports have indicated the involvement of lysosome-related proteins in the macrophage defence response to MTB and MB infection (van der Wel et al., 2007; Bach et al., 2008). Interestingly, many immunity-related proteins associated with molecular function category terms were activated in response to MTB and MB infection; the enriched terms included NADPH-haemoprotein reductase activity, interleukin-1 receptor binding, scavenger receptor activity, calcium-dependent protein binding, and cytokine activity (**Figure 3B**; **Table 3**). When attacked by pathogenic bacteria, calcium-related signalling pathways are rapidly activated to produce a defence response, including an inflammatory reaction. Importantly, within the biological process category, numerous defence-related proteins were significantly triggered by both types of pathogenic bacterial infections; the enriched terms included acute-phase response, cellular response to molecule of bacterial origin, cellular response to biotic stimulus, response to molecule of bacterial origin, acute inflammatory response, inflammatory response, defence response, response to bacterium, cellular response to oxygen-containing compound, regulation of antimicrobial humoral response, positive regulation of antimicrobial humoral response, response to biotic stimulus, and regulation of inflammatory response (**Figure 3C**; **Table 3**). In summary, we concluded that the terms related to autophagy such as inflammation-related processes and defences against pathogenic bacteria represent critical responses of BAMs challenged with MTB or MB infection.

**Table 3:** Significantly enriched Gene Ontology terms in both MTB- and MB-infected bovine alveolar macrophages.

GO ID	Description	Gene Number	*P* value	Genes
**GO:0043202**	lysosomal lumen	1	0.010493	A0A3S5ZPN6
**GO:0000323**	lytic vacuole	2	0.030988	A0A3S5ZPN6; P09428
**GO:0005764**	lysosome	2	0.030988	A0A3S5ZPN6; P09428
**GO:0005776**	autophagosome	1	0.048991	P09428
**GO:0003958**	NADPH-haemoprotein reductase activity	1	0.009069	F1MYR5
**GO:0005149**	interleukin-1 receptor binding	1	0.012076	P09428
**GO:0005044**	scavenger receptor activity	1	0.018066	A0A3S5ZPN6
**GO:0048306**	calcium-dependent protein binding	1	0.026989	F1MX83
**GO:0005125**	cytokine activity	1	0.041702	P09428
**GO:0006953**	acute-phase response	2	1.04E-05	F1MNI5; P09428
**GO:0071219**	cellular response to molecule of bacterial origin	3	1.87E-05	F1MGW6; F1MYR5; P09428
**GO:0071216**	cellular response to biotic stimulus	3	3.27E-05	F1MGW6; F1MYR5; P09428
**GO:0002237**	response to molecule of bacterial origin	3	7.37E-05	F1MGW6; F1MYR5; P09428
**GO:0002526**	acute inflammatory response	2	9.64E-05	F1MNI5; P09428
**GO:0006954**	inflammatory response	3	0.0003	F1MNI5; F1MYR5; P09428
**GO:0006952**	defence response	4	0.000312	F1MGW6; F1MNI5; F1MYR5; P09428
**GO:0009617**	response to bacterium	3	0.000534	F1MGW6; F1MYR5; P09428
**GO:1901701**	cellular response to oxygen-containing compound	3	0.001038	F1MGW6; F1MYR5; P09428
**GO:0002759**	regulation of antimicrobial humoral response	1	0.002037	F1MGW6
**GO:0002760**	positive regulation of antimicrobial humoral response	1	0.002037	F1MGW6
**GO:0009607**	response to biotic stimulus	3	0.002496	F1MGW6; F1MYR5; P09428
**GO:0050727**	regulation of inflammatory response	2	0.00301	F1MNI5; F1MYR5

“P < 0.05”.

To delve deeper into the roles of the upregulated proteins in response to MTB and MB infection, we performed a more detailed annotation of the nine aforementioned significantly upregulated proteins in MTB- and MB-infected BAMs. To this end, we performed a KEGG pathway analysis with a significance level threshold set at p < 0.05 with animal reference pathways from the KEGG database (**Figure 3D**). The upregulated proteins quantified via label-free methods following infection by either pathogenic bacterium types were primarily enriched in pathways such as the NF-kappa B signalling pathway, IL-17 signalling pathway, C-type lectin receptor signalling pathway, tuberculosis, cytokine–cytokine receptor interaction, inflammatory mediator regulation of TRP channels, Toll-like receptor signalling pathway, peroxisome, and HIF-1 signalling pathway (**Figure 3D**). These pathways are primarily activated in the inflammatory and immunity-related responses of host cells attacked by pathogenic microbes. Notably, the NF-kappa B signalling pathway, IL-17 signalling pathway, and Toll-like receptor signalling pathway play crucial roles in TB infection. Therefore, we highlight that both MTB and MB infection separately elicited inflammatory responses in BAMs.

### Functional analysis of upregulated proteins specifically, activated by MTB infection

**Figure 4:**
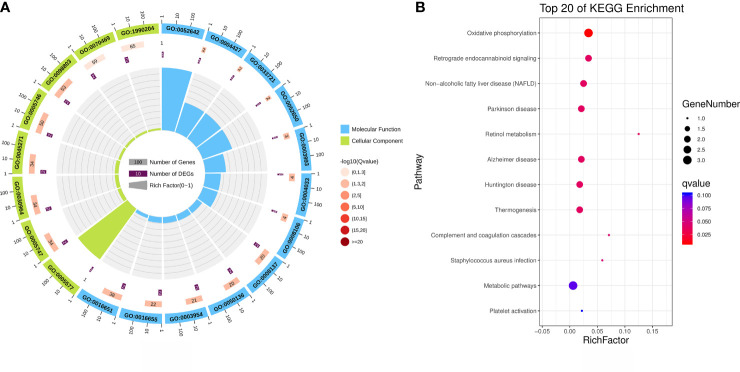
Functional annotation analysis of the upregulated proteins that were activated only after MTB infection. **(A)** GO category analysis of the upregulated proteins associated with MTB infection. The proteins were categorised according to GO annotation terms, and the number of proteins enriched in cellular component (green) and molecular function (blue) categories is shown. The number of proteins in each GO category is displayed for each term. The colour of each column represents the significance of each enriched category. **(B)** Pathway enrichment analysis of upregulated proteins in MTB-infected BAM cells. The scatter plot shows the pathway impact and enrichment results for all significantly upregulated proteins. Each point represents a different metabolic pathway. The various colour intensities indicate different levels of significance of the metabolic pathways from low (blue) to high (red). The size of each point represents the number of proteins involved in each corresponding metabolic pathway.

GO analysis offers a universally recognised set of identifiers to describe the attributes of proteins within an organism. Thus, we applied GO functional annotation analysis to the upregulated proteins exclusively expressed via MTB infection to elucidate the specific responses of BAMs after MTB challenge (**Figure 4A**; **Table 4**). All eight proteins expressed after MTB infection were simultaneously annotated within GO categories. The three classical GO categories - molecular function, biological process, and cellular component - were extensively covered by these proteins (**Figure 4A**; **Table 4**). Our results showed that the terms related to molecular function and cellular component were the main terms significantly enriched in MTB-infected BAMs (**Figure 4A**; **Table 4**). Fourteen terms were significantly enriched in the cellular component category, including fibrinogen complex, mitochondrial respiratory chain complex I, NADH dehydrogenase complex, respiratory chain complex I, mitochondrial respiratory chain, respiratory chain complex, respiratory chain, oxidoreductase complex, photoreceptor inner segment, inner mitochondrial membrane protein complex, mitochondrion, mitochondrial protein complex, mitochondrial part, and mitochondrial membrane part (**Figure 4A**; **Table 4**). The results further showed that all significantly enriched cellular component-related terms were involved in energy metabolism and functioned in mitochondria, suggesting that MTB infection triggers significant changes in the energy metabolism of BAMs, which may facilitate defence responses against pathogenic bacterial attacks by BAMs. Similarly, in the molecular function category, terms related to energy metabolism were also enriched as the related proteins in BAM responded to MTB infection. These terms included NADH dehydrogenase activity, oxidoreductase activity acting on NAD(P)H, lysophosphatidic acid phosphatase activity, inorganic diphosphatase activity, aldehyde dehydrogenase (NADP+) activity, and NADP-retinol dehydrogenase activity (**Figure 4A**; **Table 4**). Therefore, we propose that when challenged by MTB infection, BAMs may show accelerated energy metabolism to counteract MTB assaults, thereby safeguarding the organism.

**Table 4:** Significantly enriched categories relevant to the molecular function of eight MTB-specifically activated proteins.

GO ID	Description	Gene Number	*P* value	Genes
**GO:0008137**	NADH dehydrogenase (ubiquinone) activity	2	0.000887	P23935; P42028
**GO:0050136**	NADH dehydrogenase (quinone) activity	2	0.000887	P23935; P42028
**GO:0003954**	NADH dehydrogenase activity	2	0.00098	P23935; P42028
**GO:0016655**	oxidoreductase activity, acting on NAD(P)H	2	0.001076	P23935; P42028
**GO:0052642**	lysophosphatidic acid phosphatase activity	1	0.002358	A6H757
**GO:0016651**	oxidoreductase activity, acting on NAD(P)H	2	0.003218	P23935; P42028
**GO:0004427**	inorganic diphosphatase activity	1	0.004711	Q2KIV7
**GO:0033721**	aldehyde dehydrogenase (NADP+) activity	1	0.004711	E1BM93
**GO:0052650**	NADP-retinol dehydrogenase activity	1	0.004711	E1BM93
**GO:0003993**	acid phosphatase activity	1	0.007059	A6H757
**GO:0004033**	aldo-keto reductase (NADP) activity	1	0.009402	E1BM93
**GO:0008106**	alcohol dehydrogenase (NADP+) activity	1	0.009402	E1BM93
**GO:0016491**	oxidoreductase activity	3	0.017809	E1BM93; P23935; P42028
**GO:0003746**	translation elongation factor activity	1	0.030281	P43896
**GO:0051539**	4 iron, 4 sulfur cluster binding	1	0.037156	P42028
**GO:0016620**	oxidoreductase activity, acting on NADP as acceptor	1	0.043989	E1BM93

“P < 0.05”.

We further analysed the KEGG database to determine the biological process and terms enriched by the eight proteins activated by MTB infection (**Figure 4B**). All eight proteins were predominantly associated with energy metabolism, represented by oxidative phosphorylation, and the immune system, as evidenced by complement and coagulation cascades (**Figure 4B**). Moreover, the significantly enriched process identified in response to pathogenic bacteria was *Staphylococcus aureus* infection (**Figure 4B**). In conclusion, under the challenge by MTB infection, MABs showed a marked increase in energy metabolism, which appeared to be the primary response to counter MTB assaults.

### Functional analysis of upregulated proteins specifically, activated by MB infection

**Figure 5:**
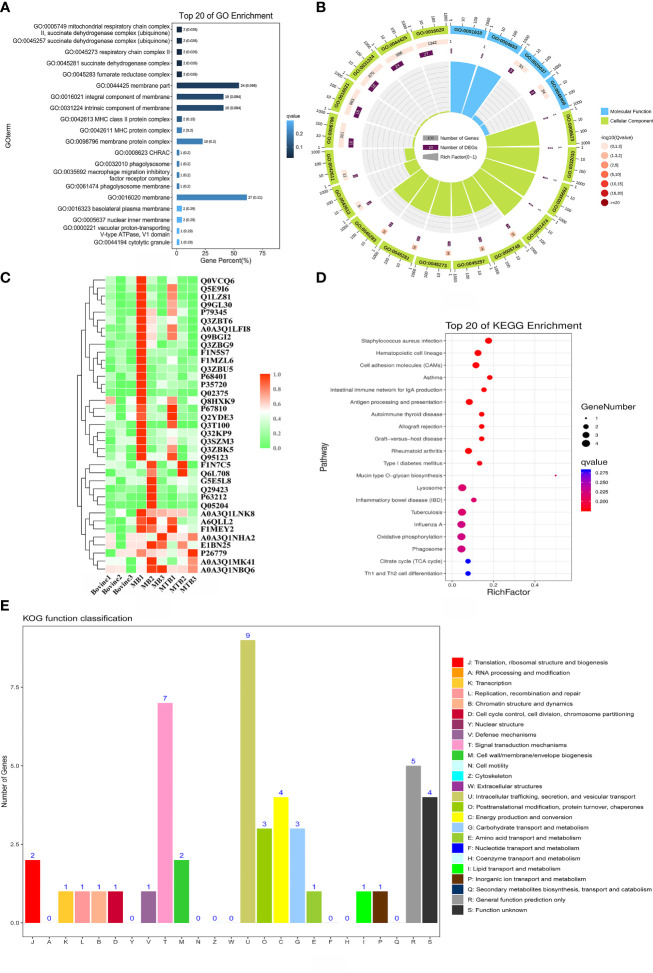
Functional annotation analysis of significantly upregulated proteins associated with MB infection. **(A, B)** GO enrichment analysis for 51 upregulated proteins in BAMs after MB infection challenge. The proteins were categorised according to GO annotation terms, and the number of proteins in the cellular component **(A)**, biological process and molecular function **(B)** categories is shown. The number of proteins involved in each GO category is displayed by enriched term. The colour of each column represents the significance of each enriched category. **(C)** Clustering analysis of proteins differentially expressed in the three treatment groups. Green indicates proteins with relatively low expression in the corresponding samples, whereas red indicates relatively high expression abundance. **(D)** The pathway enrichment results based on these MB-induced upregulated proteins in BAMs. The scatter plot shows the pathway impact and enrichment results for all 51 significantly upregulated proteins. Each point represents a different metabolic pathway. The various colour intensities indicate different levels of significance of the metabolic pathways from low (blue) to high (red). The number of proteins involved in each pathway is shown by the size of each corresponding point. **(E)** Functional classification of proteins as determined by eukaryotic orthologous group (KOG) analysis. The number of proteins in each KOG category is shown at the top of each column. J: translation, ribosomal structure and biogenesis; A: RNA processing and modification; K: transcription; L: replication, recombination and repair; B: chromatin structure and dynamics; D: cell cycle control, cell division, chromosome partitioning; Y: nuclear structure; V: Defence mechanisms; T: signal transduction mechanisms; M: cell wall/membrane/envelope biogenesis; N: cell motility; Z: cytoskeleton; W: extracellular structures; U: intracellular trafficking, secretion, and vesicular transport; O: posttranslational modification, protein turnover, chaperones; C: energy production and conversion; G: carbohydrate transport and metabolism; E: amino acid transport and metabolism; F: nucleotide transport and metabolism; H: coenzyme transport and metabolism; I: lipid transport and metabolism; P: inorganic ion transport and metabolism; Q: secondary metabolite biosynthesis, transport and catabolism; R: general function prediction only; S: function unknown.

In the current study, we aimed to identify proteins exclusively associated with MB infection in BAMs. We compared protein expression profiles from three treatments: MTB infection, MB infection, and normal (uninfected) BAMs. Hierarchical clustering of these upregulated proteins revealed similar trends. All 51 significantly upregulated proteins showed increased expression exclusively in BAMs infected with MB (**Figure 5C**; **Table 2**).

Subsequently, we performed GO enrichment analysis on all the significantly upregulated proteins to elucidate the biological processes affected during the progression of MB infection (**Figures 5A, B**; **Table 5**). For the cellular component category, several terms relevant to energy metabolism, autophagy, and inflammation were significantly enriched. These terms included mitochondrial respiratory chain complex II, succinate dehydrogenase complex (ubiquinone), respiratory chain complex II, phagolysosome, macrophage migration inhibitory factor receptor complex, phagolysosome membrane, autolysosome, NLRP3 inflammasome complex, AIM2 inflammasome complex, and autophagosome (**Figure 5A**; **Table 5**). This finding suggested that MB infection induced various changes in energy metabolism, autophagy, and inflammation of BAM. Additionally, three molecular function terms related to oxidation–reduction reactions, including peroxidase activity, oxidoreductase activity and antioxidant activity, were enriched for the 51 upregulated proteins (**Figure 5B**; **Table 5**). Peroxidase activity, oxidoreductase activity and antioxidant activity perform important functions in BAMs to defend against pathogenic bacterial attacks, such as superoxide dismutase A (Shah et al., 2015). Notably, in the biological process category, several terms associated with energy metabolism, autophagy, defence response to bacteria and immune response were markedly enriched in the group of BAMs with MB infection; these terms included defence response, hydrogen peroxide-mediated programmed cell death, immune response, mitochondrial electron transport, succinate to ubiquinone, negative regulation of inflammatory response, negative regulation of response to external stimulus, regulation of natural killer cell-mediated immunity, respiratory burst after phagocytosis and response to wounding (**Figure 5B**; **Table 5**). Collectively, these GO enrichment analyses suggested that the primary responses of BAMs under pathogenic bacterial MB attack involve the activation of energy metabolism pathways, autophagy, the defence response to bacteria, and immune response.

**Table 5:** Significantly enriched Gene Ontology terms only in MB-infected bovine alveolar macrophages.

GO ID	Description	out (44)	*P* value
**GO:0016209**	antioxidant activity	2	0.041502
**GO:0044754**	autolysosome	1	0.042823
**GO:0005776**	autophagosome	1	0.242651
**GO:0016338**	calcium-independent cell–cell adhesion via plasma membrane cell-adhesion molecules	1	0.014941
**GO:0006952**	defence response	7	0.030843
**GO:0008626**	granzyme-mediated apoptotic signalling pathway	1	0.014941
**GO:0010421**	hydrogen peroxide-mediated programmed cell death	1	0.029663
**GO:0006955**	immune response	7	0.043833
**GO:0036481**	intrinsic apoptotic signalling pathway in response to hydrogen peroxide	1	0.029663
**GO:0006121**	mitochondrial electron transport, succinate to ubiquinone	2	0.001285
**GO:0005749**	mitochondrial respiratory chain complex II	2	0.000609
**GO:1900016**	negative regulation of cytokine production involved in inflammatory response	1	0.04417
**GO:0050728**	negative regulation of inflammatory response	2	0.022926
**GO:0032102**	negative regulation of response to external stimulus	3	0.016975
**GO:0072559**	NLRP3 inflammasome complex	1	0.042823
**GO:0004601**	peroxidase activity	2	0.017343
**GO:0032010**	phagolysosome	1	0.014478
**GO:0061474**	phagolysosome membrane	1	0.014478
**GO:0002717**	positive regulation of natural killer cell mediated immunity	1	0.014941
**GO:0097468**	programmed cell death in response to reactive oxygen species	1	0.029663
**GO:0002715**	regulation of natural killer cell mediated immunity	1	0.014941
**GO:1903034**	regulation of response to wounding	4	0.010858
**GO:0045728**	respiratory burst after phagocytosis	1	0.029663
**GO:0045273**	respiratory chain complex II	2	0.000609
**GO:0042060**	wound healing	5	0.000627

“P < 0.05”.

The Cluster of Orthologous Groups (COG) of proteins database is instrumental in understanding protein functions due to their roles as indicated by orthologous classification. To identify the specific functions of the 51 upregulated proteins, we analysed them using the COG protein database. The results showed that these proteins were mainly enriched in 17 of the 25 COG categories (**Figure 5E**). The most frequently enriched COG terms were signal transduction mechanisms, intracellular trafficking, secretion, and vesicular transport, and energy production and conversion (**Figure 5E**). Furthermore, the term defence mechanisms was also enriched for these MB-activated proteins (**Figure 5E**). In addition, several identified proteins were enriched in posttranslational modification, protein turnover, chaperones and carbohydrate transport and metabolism. These findings suggested that these functional classifications also engage in some BAM responses to MB infection (**Figure 5E**).

Further analysis of the aforementioned 51 proteins using the KEGG pathway database provided insights into the principal biochemical metabolism and signal transduction pathways activated in BAMs in response to MB infection (**Figure 5D**). The results showed that all significant annotated proteins were mapped to 18 KEGG pathways, notably lysosome, tuberculosis, phagosome, apoptosis, the mTOR signalling pathway, and autophagy (**Figure 5D**). Interestingly, TB, a disease caused by MB infection, was also identified as significant with a P value of 0.032 (**Figure 5D**). Additionally, autophagy and apoptosis were major terms in the biological process category, signifying their important function in the defence response of BAMs to pathogenic bacterial MB. Lysosomes and the mTOR signalling pathway perform important functions in the defence of macrophages against MTB and MB infection.

In conclusion, we found that MB infection altered the expression of proteins involved in energy metabolism, autophagy, apoptosis, lysosomal functions, and inflammation. The activation of these processes might constitute the primary defensive programs triggered by BAM to counteract pathogenic MB attacks.

### MB infection-induced proteins involved in the complex interaction network in bovine alveolar macrophages

**Figure 6:**
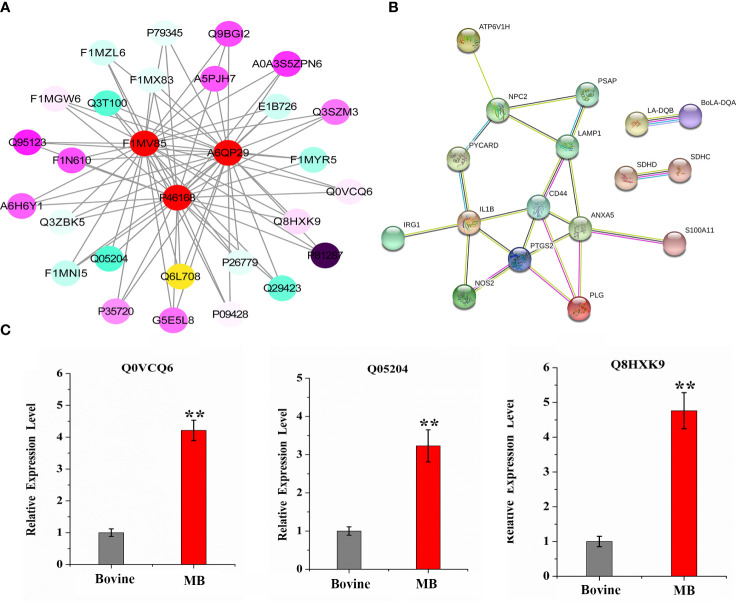
Complex interaction and coexpression network of key MB-altered proteins. **(A)** Global protein interaction network analysis of significantly differentially expressed bovine proteins after MB infection enriched in key pathways and GO terms. All the key differentially expressed proteins were submitted to the STRING tool (http://string.embl.de/) and the protein–protein interaction network was thus predicted. **(B)** Coexpression network of significantly differentially expressed bovine proteins after MB infection that are enriched in key pathways and GO terms. The proteins shown in the red circle represent three downregulated proteins that were suppressed by MB infection in BAMs. The various colour intensities display the correlation level with the linked proteins from negative (blue) to positive (purple). **(C)** qRT–PCR verification of important defence- and autophagy-related proteins expression induced by MB infection. The relative expression levels are represented by the fold change of the value obtained via the 2^-△△Ct^ method. (**P < 0.01).

Based on the analyses performed, 29 proteins were identified as crucial proteins activated in BAMs in response to MB infection (**Table 2**). Among these proteins, 26 proteins were upregulated during MB infection, while only three proteins — F1MV85, P46168 and A6QP29 — were downregulated in BAMs after MB challenge (**Table 2**). Additionally, the expression of three proteins relevant to defence and autophagy was validated by qRT–PCR. The results indicated that the genes encoding these proteins were significantly upregulated in macrophages following MB infection, further supporting their crucial roles of these proteins in defending BAMs against MB infection (**Figure 6C**). In this study, we utilised STRING (version 10; Szklarczyk et al., 2011), a database that provides known and predicted PPIs, to obtain additional protein information for subsequent functional validation of the key proteins activated by MB infection (**Figure 6A**). Notably, the results highlighted numerous PPIs among the 29 key proteins (**Figure 6A**). Under the conditions described, all proteins were interconnected with the downregulated proteins (**Figure 6A**). In contrast, in the subsequently produced coexpression network, we observed a clear negative correlation between the downregulated proteins and upregulated proteins, forming complex links between them (**Figure 6B**). These proteins were mainly involved in various energy metabolism-, autophagy-, and immunity-related pathways, including tuberculosis, lysosome, phagosome, Th17 cell differentiation, and oxidative phosphorylation (**Table 6**). We inferred that a complex protein network is formed, with the expression of proteins altered in BAMs in response to MB infection to initiate defence responses.

**Table 6:** Key pathways of proteins activated by MB infection.

#Term ID	Term description	Gene count	False discovery rate
**bta05152**	Tuberculosis	6	4.27E-06
**bta04142**	Lysosome	4	0.00022
**bta04145**	Phagosome	4	0.0005
**bta04659**	Th17 cell differentiation	3	0.0024
**bta00190**	Oxidative phosphorylation	3	0.0035
**bta04672**	Intestinal immune network for IgA production	2	0.0081
**bta04657**	IL-17 signalling pathway	2	0.0163
**bta04064**	NF-kappa B signalling pathway	2	0.0181
**bta04668**	TNF signalling pathway	2	0.0205

“P < 0.05”

## Discussion

The precise mechanisms underlying mycobacterium and host adaptation during infection remains unknown. MTB, a human pathogen, is exceptionally adapted for human-to-human transmission (Daniela Brites, 2015), and although it is sporadically isolated from cattle (Ocepek et al., 2005; Romha et al., 2018) and other animals in close contact with humans, it is infrequently transmitted between animal populations (Whelan et al., 2010; Behr and Gordon, 2015). There is accumulating evidence to suggest that human MTB is nontoxic or that infection with MTB is attenuated in cattle (Whelan et al., 2010; Villarreal-Ramos et al., 2018). MB, in contrast, is seldom transmitted to humans but can occasionally infect humans, primarily through the consumption of raw milk or close contact with infected cattle (Muller et al., 2013). Transmission of MB, has also been reported in immunocompetent humans, but these reports are rare (Blázquez et al., 1997). However, infection of human hosts by MB can potentially lead to TB-related fatalities (Muller et al., 2013). Previous research showed that both MTB or MB infection can provoke responses in both cattle and humans, particularly in macrophages from these hosts (Daley, 2010). Cells can eliminate mycobacteria in MB- and MTB-colonised tissues by undergoing apoptosis or autophagy. However, differences in the responses of macrophages to MTB and MB were unclear. In this study, we carried out an in-depth proteomic analysis of macrophages infected with MTB or MB, concluding that macrophages respond more robustly to MB infection than to MTB infection. During the infection process, hosts infected with MTB showed increased energy-related mechanistic activity, which may lead to a more efficient defence against MB and MTB infection. Moreover, macrophages showed increased autophagy and inflammation-related activities to defend against MB infection, while the response to MTB infection involved the activation of only 8 proteins, which were associated with lysophosphatidic acid phosphatase activity, oxidoreductase activity, and aldehyde dehydrogenase (NADP+) activity. Moreover, we discovered that various signalling pathways associated with autophagy and inflammation-related processes were affected by MB infection, potentially contributing to the macrophage defence against MB attack. Collectively, our findings provide novel insights into the distinct mechanisms underpinning the critical interactions of MB and MBT with macrophages.

The interaction between MB and macrophages results in chronic inflammatory and autophagy-related responses, which inhibit mycobacterial growth (Moraco and Kornfeld, 2014). Autophagy, an essential program in macrophages that eliminates MB bacteria ensures that autophagic cells do not release intracellular MTB or MB bacterial components. As MTB is ingested via phagocytosis, antigenic sites are exposed, and toxic substances are released. In response, the stimulated macrophages secrete cytokines and chemokines, thereby initiating the innate immune response (Pieters, 2008). Macrophages can degrade phagocytosed MTB through acidic hydrolase after phagosomes fuse with lysosomes, thereby killing or inhibiting intracellular MTB growth. In contrast, ingested MTB can hinder phagocytosis-based acidification and the fusion of phagosomes and lysosomes, enabling MTB to avoid proteolytic enzyme hydrolysis and the induction of subsequent immune responses; this is a key strategy of MTB evasion from host cell clearance (van der Wel et al., 2007; Bach et al., 2008). Thus, the expression of lysosome-associated genes is vital for host elimination of pathogens. In our study, we discovered that MTB infection activated autophagy-associated proteins in macrophages and that MB infection influenced the expression of these proteins to an even greater extent. Moreover, we found that infection with either MTB or MB substantially triggered the expression of host lysosome-associated proteins, although MTB infection exerted a more significant effect. “We propose that M. bovis may inhibit the expression of lysosomal and autophagy-related proteins, such as F1MV85, P46168, and A6QP2 (Toyofuku et al., 2015; Peng et al., 2022)9, diminish the fusion of phagosomes and lysosomes, and consequently weaken the host autophagy response to effectively infect host cells. This mechanism might contribute to bacteria evasion from the host macrophage immune responses. Hosts exhibit varied gene regulatory responses to MTB infection, encompassing mechanisms such as long noncoding RNAs (lncRNAs), miRNAs, and splicing events (Kumar et al., 2016; Pawar et al., 2016; Li et al., 2022). Notably, both lncRNAs and miRNAs influence autophagy-related signalling pathways, serving as pivotal players in modulating MTB immunity (Ouimet et al., 2016; Zhang et al., 2022). In our recent experiments, we observed a more pronounced autophagic response in alveolar macrophages infected with MB compared to those infected with MTB. Based on these findings, we posit that alterations in lncRNA and miRNA expressions might be central to the differential host responses towards MTB and MB infections. This intriguing interplay will be the centerpiece of our forthcoming research.

In conclusion, our findings suggest that defence-related genes respond to MB infection through complex signalling networks, including the NF-κB signalling pathway, IL-17 signalling pathway, cytokine–cytokine receptor interaction, inflammatory mediator regulation of TRP channels, Toll-like receptor signalling pathway, and HIF-1 signalling pathway. The TLR-2 pathway plays a regulatory function in immunomodulation through the export of agonists (Prados-Rosales et al., 2011; Athman et al., 2015).. In parallel, recent studies have revealed that IL-17 is important for proinflammatory responses and chemokine regulation, with a robust IL-17 response correlating with the severity of human diseases. *In vitro* and *in vivo* studies, the generation of Th17 cells led to higher IL-17 expression (Liang et al., 2006). Notably, among the signalling pathways involved in these cells, the NF-κB and Toll-like receptor signalling pathways were closely associated with the activation of the host defence against MB infection; this reaction included various proinflammatory responses and the release of antimicrobial effectors (Pilli et al., 2012; Jia et al., 2015; Liu et al., 2018). Previous studies have demonstrated that MB infection can stimulate the production of TNF-α`in RAW264.7 cells and activate the NF-κB pathway through TLR2-mediated signal transduction, which play pivotal roles in autophagy- and inflammation-related progression (Pilli et al., 2012; Liu et al., 2018).. In contrast, the proteomic profiles of MTB-infected BAMs did not exhibit enrichment in these signalling pathways. It has been reported that MTB infection primarily suppresses the MAPK signalling pathway and lipid metabolism pathway in macrophages, which are critical for regulating the MTB-induced expression of immunoregulatory molecules, such as TNF-α and IL-17 (Shukla et al., 2018). In addition, one study illustrated that MB can trigger autophagy- and inflammation-related progression primarily via the NF-κB signalling pathway, IL-17 signalling pathway, cytokine–cytokine receptor interaction, inflammatory mediator regulation of TRP channels, and the Toll-like receptor signalling pathway. This finding enhances our understanding of the MB-mediated innate immune response triggered by MB–macrophage interactions.

In conclusion, our research provides substantial evidence showing that macrophages can induce different responses to combat infections with MTB and MB, and these responses involves a variety of autophagy- and inflammation-related processes and signalling pathways. Moreover, our findings suggest that MB may suppress autophagy- and inflammation-related protein and signalling pathway activity to evade host cell clearance programs and facilitate bacterial colonization within the host. The results of our study offer valuable insights into the pathogenesis of TB, highlighting 29 proteins uniquely induced by MB infection and another 8 proteins selectively upregulated during MTB infection. These proteins hold promise as novel biomarkers, potentially paving the way for the development of enhanced therapeutic strategies. Furthermore, our data may help inform strategies to disrupt the cross-species transmission of MB, which has significant implications for public health.

## Data availability statement

The authors acknowledge that the data presented in this study must be deposited and made publicly available in iProX repository, accession number: IPX0006970000.

## Author contributions

YC: Conceptualization, Data curation, Formal Analysis, Investigation, Methodology, Project administration, Validation, Visualization, Writing – original draft. WG: Investigation, Methodology, Visualization, Formal Analysis, Writing – original draft, Writing – review & editing. PW: Supervision, Validation, Data curation, Writing – review & editing. GZ: Visualization, Conceptualization, Writing – review & editing. XW: Resources, Formal Analysis, Investigation, Writing – review & editing. LJ: Supervision, Data curation, Writing – review & editing. JZ: Conceptualization, Writing – review & editing. YW: Funding acquisition, Resources, Writing – review & editing. ZW: Writing – review & editing. YL: Funding acquisition, Project administration, Resources, Visualization, Writing – review & editing.
